# Supplementary material for: Multiple thresholds and trajectories of microbial biodiversity predicted across browning gradients by neural networks and decision tree learning
Source: ISME Commun. 2021 Aug 16;1:37. doi: 10.1038/s43705-021-00038-8 (PMC9723588; doi:10.1038/s43705-021-00038-8)
Supplement: Supplementary file 10 — Supplementary Table S1 [file 43705_2021_38_MOESM10_ESM.pdf]

Supplementary Table S1. Complete list of environmental variables and summary statistics.

	Latitude	Longitude	Altitude	Area	Depth	Temperature	Secchi	O2	CO2	CH4	N2O
	Min. :58.76	Min. : 5.402	Min. : 7.0	Min. : 1.09	Min. : 3.50	Min. : 9.93	Min. : 1.000	Min. :0.6418	Min. :0.04085	Min. : 4.193	Min. :0.9911
	1st Qu.:59.59	1st Qu.: 9.909	1st Qu.: 72.0	1st Qu.: 2.04	1st Qu.:10.00	1st Qu.:16.34	1st Qu.: 2.000	1st Qu.:0.8338	1st Qu.:1.41620	1st Qu.: 16.712	1st Qu.:1.1326
	Median :60.15	Median :11.626	Median :163.0	Median : 3.40	Median :17.00	Median :17.97	Median : 2.500	Median :0.8750	Median :1.74044	Median : 24.484	Median :1.2162
	Mean :60.25	Mean :11.515	Mean :191.2	Mean : 12.70	Mean :20.77	Mean :17.69	Mean : 3.255	Mean :0.8569	Mean :2.25768	Mean : 40.793	Mean :1.2887
	3rd Qu.:60.82	3rd Qu.:14.420	3rd Qu.:260.0	3rd Qu.: 13.66	3rd Qu.:25.00	3rd Qu.:19.59	3rd Qu.: 3.938	3rd Qu.:0.9007	3rd Qu.:2.53373	3rd Qu.: 49.583	3rd Qu.:1.3271
	Max. :62.53	Max. :18.521	Max. :540.0	Max. :140.09	Max. :76.00	Max. :21.41	Max. :12.500	Max. :0.9908	Max. :6.47866	Max. :260.597	Max. :4.2042
	NA	NA	NA	NA	NA's :4	NA's :1	NA's :1	NA's :1	NA's :1	NA's :1	NA's :1

	pH	TIC	TOC	TN	TP	NO3	SiO2	PO4	PON	POC	POP
	Min. :5.410	Min. :0.07061	Min. : 0.3441	Min. :0.08657	Min. : 0.500	Min. : 0.00	Min. : 224	Min. :-1.000	Min. : 11.85	Min. : 68.0	Min. : 1.900
	1st Qu.:6.830	1st Qu.:0.77268	1st Qu.: 3.7675	1st Qu.:0.23280	1st Qu.: 3.000	1st Qu.: 8.20	1st Qu.:1787	1st Qu.:-1.000	1st Qu.: 30.05	1st Qu.: 198.0	1st Qu.: 3.000
	Median :7.035	Median :1.32950	Median : 6.5250	Median :0.29800	Median : 4.550	Median : 65.65	Median :3021	Median : 1.000	Median : 41.21	Median : 300.0	Median : 4.100
	Mean :7.027	Mean :1.81232	Mean : 6.2133	Mean :0.41236	Mean : 6.046	Mean : 166.59	Mean :2984	Mean : 1.494	Mean : 61.40	Mean : 391.9	Mean : 5.058
	3rd Qu.:7.215	3rd Qu.:1.92625	3rd Qu.: 7.6190	3rd Qu.:0.42850	3rd Qu.: 7.250	3rd Qu.: 193.70	3rd Qu.:4037	3rd Qu.: 3.000	3rd Qu.: 64.02	3rd Qu.: 381.0	3rd Qu.: 5.400
	Max. :8.875	Max. :9.18700	Max. :12.8950	Max. :1.52600	Max. :27.450	Max. :1153.35	Max. :5776	Max. :16.000	Max. :491.77	Max. :3371.0	Max. :16.900
	NA	NA's :1	NA	NA	NA	NA	NA	NA	NA	NA	NA

	Chla	APA	a.phyto.m	a.dom.m	a.tripton.m	tot.phyto	f.hetero	esd	C.P..at.	C.P.calc	dom_level
	Min. : 0.770	Min. : 0.01	Min. :0.02000	Min. :0.190	Min. :0.0100	Min. :0.09018	Min. :0.0008209	Min. : 6.872	Min. : 49.3	Min. : 48.8	L:28
	1st Qu.: 2.120	1st Qu.: 12.71	1st Qu.:0.04000	1st Qu.:0.630	1st Qu.:0.0600	1st Qu.:0.20683	1st Qu.:0.0173802	1st Qu.: 8.920	1st Qu.:142.4	1st Qu.:144.2	M:49
	Median : 2.705	Median : 25.45	Median :0.06000	Median :1.020	Median :0.1000	Median :0.28579	Median :0.0350892	Median :10.294	Median :175.6	Median :175.2	NA
	Mean : 4.501	Mean : 34.81	Mean :0.07493	Mean :1.195	Mean :0.1284	Mean :0.54382	Mean :0.0371366	Mean :11.091	Mean :187.6	Mean :187.6	NA
	3rd Qu.: 4.518	3rd Qu.: 36.23	3rd Qu.:0.08000	3rd Qu.:1.530	3rd Qu.:0.1800	3rd Qu.:0.51783	3rd Qu.:0.0471961	3rd Qu.:12.670	3rd Qu.:213.2	3rd Qu.:212.0	NA
	Max. :29.480	Max. :401.02	Max. :0.33000	Max. :3.830	Max. :0.5100	Max. :6.56664	Max. :0.1847840	Max. :20.367	Max. :545.2	Max. :544.3	NA
	NA's :1	NA's :6	NA's :2	NA's :4	NA's :4	NA	NA	NA	NA	NA	NA
