# Supplementary material for: Multiple thresholds and trajectories of microbial biodiversity predicted across browning gradients by neural networks and decision tree learning
Source: ISME Commun. 2021 Aug 16;1:37. doi: 10.1038/s43705-021-00038-8 (PMC9723588; doi:10.1038/s43705-021-00038-8)
Supplement: Supplementary file 11 — Supplementary Table S2 [file 43705_2021_38_MOESM11_ESM.pdf]

Supplementary Table S2. Statistical comparison of linear and generalized additive models as given by the p-value, deviation explained (Dev. expl.), Akaike information criterion (AIC), generalized cross-validation (GCV), coefficient of determination (R2), the p-value of the Chi-squared test (pChi) and the number of samples (n).

	P-value	R^2	Dev. expl. [%]	GCV	AIC	pChi	n
**ACE**							
TOC	0.022/0.022	0.060/0.124	7.44/15.5	0.967/0.921	193.4/197.7	0.098	70
CDOM	0.158/0.022	0.015/0.123	2.92/15.4	1.014/0.921	201.5/194.6	0.011	70
aDOM	0.095/0.011	0.026/0.141	4.04/17.0	1.002/0.901	201.0/192.4	0.016	70
*Secchi*CO2*CH4*PO4*CDOM*		0.25/0.677	30.4/78.5	0.821/0.494	186.3/140.3	<0.001	70
**Shannon**							
TOC	0.002/0.001	0.123/0.197	13.6/22.1	0.903/0.840	193.4/188.3	0.008	70
CDOM	0.278/<0.001	0.003/0.385	1.73/44.7	1.027/0.693	202.4/174.0	<0.001	70
aDOM	0.080/<0.001	0.030/0.372	4.44/42.6	0.998/0.696	200.5/174.6	<0.001	70
*Secchi*CO2*CH4*PO4*CDOM*		0.108/0.398	17.3/50.3	0.976/0.741	198.4/176.8	<0.001	70
**Faith's**							
TOC	0.316/0.439	0/0.007	1.48/2.52	1.03/1.03	202.6/202.5	0.216	70
CDOM	0.478/0.102	-0.007/0.073	0.742/10.2	1.04/0.971	203.1/198.5	0.02	70
aDOM	0.519/0.16	-0.008/0.052	0.613/7.91	1.03/0.99	203.2/199.8	0.038	70
*Secchi*CO2*CH4*PO4*CDOM*		0.129/0.437	19.2/54.5	0.953/0.705	196.7/172.9	<0.001	70
